# Oscillatory shear stress modulates Notch-mediated endothelial mesenchymal plasticity in cerebral arteriovenous malformations

**DOI:** 10.1186/s11658-023-00436-x

**Published:** 2023-03-18

**Authors:** C. L. Karthika, Vani Venugopal, B. J. Sreelakshmi, S. Krithika, Jaya Mary Thomas, Mathew Abraham, C. C. Kartha, Arumugam Rajavelu, S. Sumi

**Affiliations:** 1 Cardiovascular Diseases and Diabetes Biology, Rajiv Gandhi Centre for Biotechnology (RGCB), Thiruvananthapuram, Kerala 695014 India; 2 Department of Neurosurgery, Sree Chitra Tirunal Institute for Medical Sciences and Technology, Thiruvananthapuram, Kerala 695011 India; 3 Department of Neurology, Amrita Institute of Medical Sciences, Amrita Vishwa Vidyapeetham, Kochi, Kerala 682041 India; 4 Department of Biotechnology, Bhupat & Jyoti Mehta School of Biosciences, Indian Institute of Technology, Madras, Chennai, Tamil Nadu 600036 India

**Keywords:** Cerebral arteriovenous malformations, Shear stress, Endothelial cells, EndMT, Notch, Small-molecule inhibitors

## Abstract

**Background:**

Cerebral arteriovenous malformations (cAVM) are a significant cause of intracranial hemorrhagic stroke and brain damage. The arteriovenous junctions in AVM nidus are known to have hemodynamic disturbances such as altered shear stress, which could lead to endothelial dysfunction. The molecular mechanisms coupling shear stress and endothelial dysfunction in cAVMs are poorly understood. We speculated that disturbed blood flow in artery–vein junctions activates Notch receptors and promotes endothelial mesenchymal plasticity during cAVM formation.

**Methods:**

We investigated the expression profile of endothelial mesenchymal transition (EndMT) and cell adhesion markers, as well as activated Notch receptors, in 18 human cAVM samples and 15 control brain tissues, by quantitative real-time PCR (qRT-PCR) and immunohistochemical evaluation. Employing a combination of a microfluidic system, qRT-PCR, immunofluorescence, as well as invasion and inhibitor assays, the effects of various shear stress conditions on Notch-induced EndMT and invasive potential of human cerebral microvascular endothelial cells (hCMEC/d3) were analyzed.

**Results:**

We found evidence for EndMT and enhanced expression of activated Notch intracellular domain (NICD3 and NICD4) in human AVM nidus samples. The expression of transmembrane adhesion receptor integrin α9/β1 is significantly reduced in cAVM nidal vessels. Cell–cell adhesion proteins such as VE-cadherin and N-cadherin were differentially expressed in AVM nidus compared with control brain tissues. Using well-characterized hCMECs, we show that altered fluid shear stress steers Notch3 nuclear translocation and promotes SNAI1/2 expression and nuclear localization. Oscillatory flow downregulates integrin α9/β1 and VE-cadherin expression, while N-cadherin expression and endothelial cell invasiveness are augmented. Gamma-secretase inhibitor RO4929097, and to a lesser level DAPT, prevent the mesenchymal transition and invasiveness of cerebral microvascular endothelial cells exposed to oscillatory fluid flow.

**Conclusions:**

Our study provides, for the first time, evidence for the role of oscillatory shear stress in mediating the EndMT process and dysregulated expression of cell adhesion molecules, especially multifunctional integrin α9/β1 in human cAVM nidus. Concomitantly, our findings indicate the potential use of small-molecular inhibitors such as RO4929097 in the less-invasive therapeutic management of cAVMs.

**Graphical Abstract:**

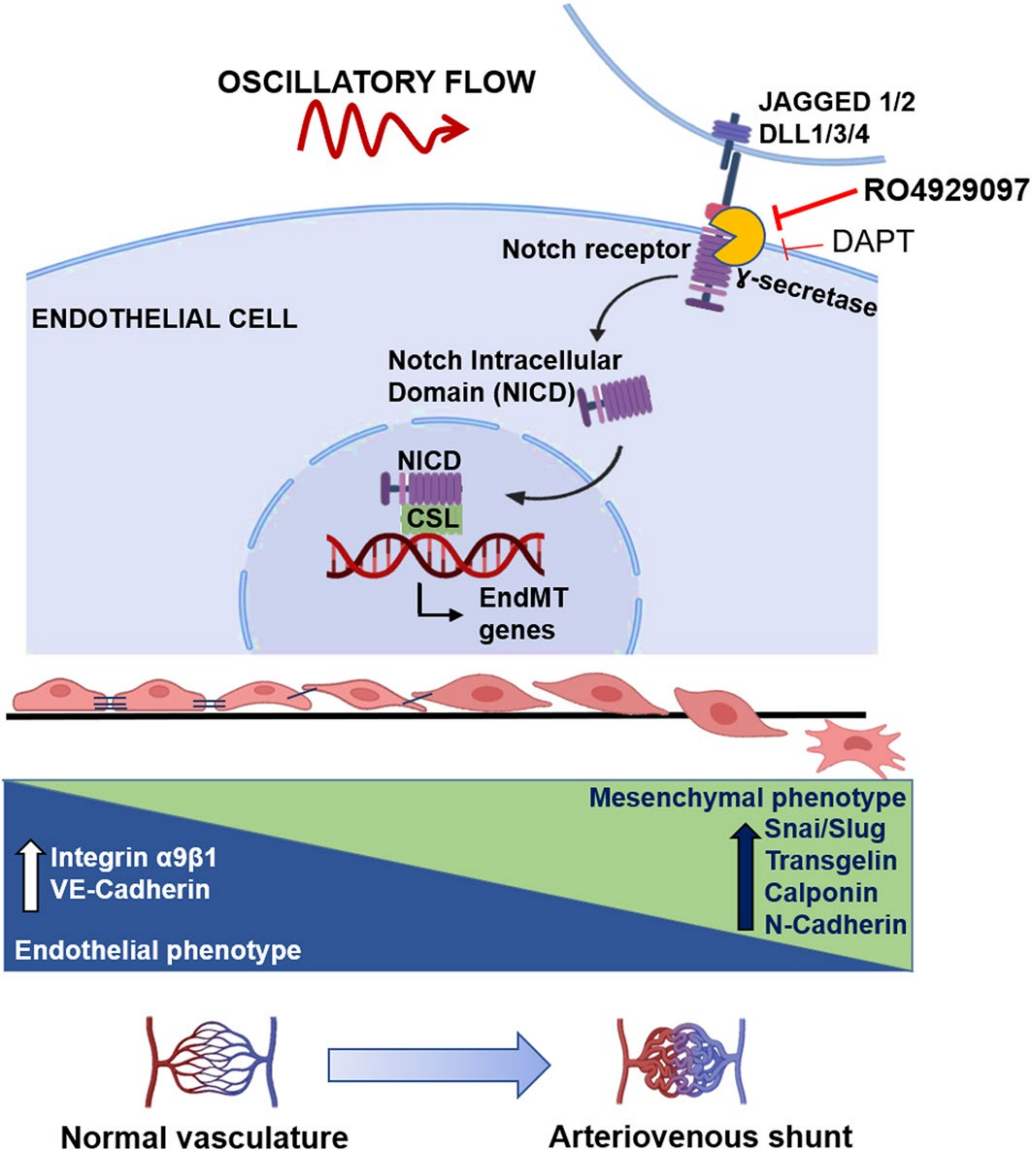

**Supplementary Information:**

The online version contains supplementary material available at 10.1186/s11658-023-00436-x.

## Background

Arteriovenous malformations (AVMs) are localized vascular anomalies resulting from direct connections between feeding arteries and draining veins, sans an intermediate capillary network. These malformations or tangles of abnormal blood vessels (nidus) can occur almost anywhere in the body. AVMs in the brain can result in extensive organ damage, secondary to seizures, and spontaneous intracranial hemorrhage [1]. Currently, corrective interventions such as surgical resection, radiosurgery, and embolization dominate the treatment algorithm of AVMs [2]. There is increasing interest to delineate the molecular pathogenesis of cerebral AVMs (cAVM) aimed to identify specific targets for pharmacological modulation of the growth of brain AVMs. However, the molecular pathogenesis of this disease is not yet delineated, due to the complicated vascular identity of cAVM nidus.

A recent study found endothelial-to-mesenchymal transition (EndMT) to contribute to AVM nidus formation [3]. The EndMT process is associated with the expression of mesenchymal markers by endothelial cells that acquire a spindle shape, as well as migratory and invasive characteristics through loss of cell adhesion properties. Nevertheless, the underlying mechanism and triggers of EndMT are vague. There is no evidence for activation of the canonical SMAD-dependent TGF-β pathway in human cAVM nidus [3]. Furthermore, factors such as disturbed blood flow and hypoxia can lead to EndMT in vascular endothelium [4, 5]. AVMs have highly altered hemodynamics because of direct arteriovenous shunts and nidus formation [6, 7]. The disturbed blood flow has been shown to have a profound influence on the endothelial layer of blood vessels [8–10]. Identifying the regulatory molecules that trigger EndMT in response to disturbed flow may provide novel insights for a pharmacological approach to the management of cAVM.

Notch signaling is a significant biological pathway that links hemodynamic mechanical cues with biochemical signaling cascades and dictates cell fate decisions [11]. Notch signaling is highly responsive to hemodynamic shear stress and regulates vasculogenesis, angiogenesis, as well as arteriovenous specification [12, 13]. Notably, both enhanced and reduced Notch signaling is associated with the development of arteriovenous shunts [14, 15]. Notch is also reported to regulate expression levels of zinc finger proteins SNAI1 and SNAI2 (Slug) in a tissue and context-dependent manner in various types of cancers [16, 17]. Activation of Notch receptors is presumed to have a role in endothelial to mesenchymal plasticity and cell invasiveness in cancers [18]. Invasiveness in the endothelial context generally causes pathological angiogenesis and AVM nidus is a site of active angiogenesis [19]. EndMT-associated invasive properties acquired by endothelial cells are closely associated with the deregulation of adhesion molecules, such as cadherins, which mediate interaction among cells, and integrins involved with cell–extracellular matrix (ECM) interactivity [20, 21].

In our present study, we observed the presence of activated Notch receptors and EndMT markers in human cAVM nidus. We also found deregulation of cell adhesion molecules in AVM nidus samples. Using in vitro fluid flow models, we provide evidence for altered shear stress-Notch3-EndMT axis in endothelial cells. Notch signaling serves as a key intermediate in sensing shear stress fluctuations and programming cells into mesenchymal characteristics. We found that altered shear stress-induced cellular invasiveness requires active Notch signaling. Furthermore, we explored the utility of gamma-secretase inhibitors (GSI), DAPT, and RO4929097 in preventing the Notch-induced EndMT and cellular invasiveness in the presence of altered shear stress.

## Methods

### Study participants

The study was approved by the human ethics committees of Rajiv Gandhi Centre for Biotechnology, Thiruvananthapuram, and collaborating hospital Sree Chitra Tirunal Institute for Medical Sciences and Technology (SCTIMST), Thiruvananthapuram. AVM tissues (*n* = 18) were collected from resected nidus of patients who underwent corrective surgery for cAVM at SCTIMST, Thiruvananthapuram, after collecting informed consent. AVMs were located in the frontal (*n* = 11), parietal (*n* = 4), and the temporal (*n* = 3) regions of the cerebrum in the recruited patients. To reduce phenotype variability, we selected only unruptured AVMs for the study. AVM was confirmed by the findings from the digital subtraction angiogram and magnetic resonance imaging (MRI) scan. Cerebral brain samples from age- and sex-matched patients (*n* = 15), who had no AVM and were operated on for temporal lobe epilepsy, were selected as control specimens for this study. Patients with cerebral aneurysms and Hereditary Hemorrhagic Telangiectasia were excluded from the current study.

### Immunohistochemistry

Immunohistochemistry was performed in formalin-fixed, paraffin-embedded tissue sections using SS Polymer-HRP IHC detection system/DAB (BioGenex, USA), as per the standardized protocol [22]. Primary antibodies, such as anti-Notch1 and anti-Notch4 (Novus Biologicals, USA), as well as anti-Notch3, anti-SNAI1/2, anti-integrin α9/β1, anti-N-cadherin, and anti-αSMA (Abcam, USA), were used. Secondary antibodies for immunohistochemistry were Goat pAb to rabbit IgG, and Rabbit pAb to mouse IgG tagged with horse radish peroxidase. DAB intensity in three random fields per slide was quantified by ImageJ software and used for histoscore analysis.

### Cell culture and characterization

For this study, we used immortalized human cerebral microvascular endothelial cells (hCMEC/d3) (Cedarlane). hCMEC/d3 were cultured in an endothelial growth medium (HiMedia Laboratories, LLC) with growth supplements, 10% FBS (Invitrogen, USA), and 1% penicillin–streptomycin cocktail (Invitrogen, USA), and the cells were maintained at 37 °C in 5% CO_2_ in a humidified chamber. hCMEC/d3 was characterized with endothelial cell marker, von Willebrand factor (vWF), as explained previously [23].

### Fluid flow-based shear stress experiments

hCMECs seeded on flow chamber μ slides [IBIDI (Integrated BioDiagnostics), Germany] were used for fluid flow-based studies. Cells seeded on μ slide, without exposure to any flow conditions, served as static control. Culture media passed through a confluent cell monolayer using the IBIDI pump system to attain parallel uniform laminar shear stress of 15 dynes/cm^2^ on hCMECs for 24 h [24]. The oscillatory flow parameter was selected for the study, considering the disturbances in blood flow in a complex nidal vascular structure with multiple feeders, shunts, and weakened drainers, as well as venous stenosis [25]. There is a disturbed flow due to the mixing of arterial and venous flow in small nidi of whole AVMs [26]. The oscillatory flow was also maintained at 15 dynes/cm^2^ and was attained in vitro by selecting the oscillating flow mode of the IBIDI Pump System. The flow rates during experiments were regulated by IBIDI pump control v1.5.4 software.

### Quantitative real-time PCR (qRT-PCR)

Total RNA from both cells exposed to all three flow conditions, as well as 18 cerebral AVM and 15 control tissues, were extracted using TRIzol (Thermo Fisher Scientific, USA) as per standard protocol. For RNA extraction, cells from six slides exposed to each flow condition were pooled together. mRNA quantification and purity were assessed by a nanodrop-1000 spectrophotometer (ThermoScientific, USA) at 260 nm. Total RNA (1 μg) was reverse transcribed using M-MLV reverse transcriptase enzyme and oligo(dT) primers (Promega, USA). cDNA was amplified with specific primers (Additional file 1: Table S1) with conditions as described previously [27] using ABI Prism 7900HT. The *GAPDH* gene was used as an internal control. For all samples, analysis was done in triplicates, and fold changes in expression levels were measured using average cycle threshold (Ct) values of all replicates.

### Immunofluorescence assay

hCMECs were exposed to static, laminar, and oscillatory shear stress conditions for 24 h. After exposure to various shear stress conditions, cells were rinsed with PBS, fixed with 4% formaldehyde for 12 min, and permeabilized with 0.1% Triton X-100 for 10 min. Slides were processed for immunostaining as previously described [23]. Primary antibodies for NICD1, 3, 4, SNAI1/2, integrin α9/β1, and N-cadherin were used. Alexa 488 and Cy3 conjugated secondary antibodies against rabbit and mouse IgG (Abcam, USA) were used to identify the signals. Details of all antibodies used in this study are given in Additional file 1: Table S2. Nuclei were counterstained with Hoechst 33342 (Sigma-Aldrich, USA). Images were captured by confocal microscope (Olympus, Japan) and mean fluorescence intensity (MFI) was quantified from five random microscopic fields using Olympus cellSens standard software. Additionally, MFI of cytoplasmic and nuclear areas of cells was assessed from three fields to ascertain the nuclear localization of proteins.

### Western blot

Total protein from tissues and cells was isolated in RIPA buffer. Transblot assays were performed as described earlier [23]. Primary antibodies used were 1:1000 dilution of Rabbit anti-NICD3 and Mouse anti-GAPDH (1:500), with Goat anti-Rabbit IgG H and L (HRP) secondary antibody (1:10,000 dilution) and Rabbit anti-Mouse IgG H and L (HRP) secondary antibody (1:10,000 dilution), respectively. Images were documented by ImageQuant LAS 500 (GE Healthcare Life Sciences, USA).

### Cell viability

MTT assay was conducted to assess cell viability at various concentrations of DAPT and RO4929097 [28]. Briefly, 10,000 cells were seeded in 96-well plates, and DAPT and RO4929097 were treated at various concentrations ranging from 1.5 µM to 0.1 µM for 24 h. Cells were incubated for 4 h using a 5 mg/ml MTT solution (20 μl) at 37 °C in the dark. The MTT-containing medium was removed, and DMSO (200 μl) was added to solubilize formazan crystals. After incubation for 1 h with shaking, the OD was measured at 570 nm using an Elisa Microplate Absorbance Reader (Robonik, India).

### Transwell invasion assay

Cell migration was assayed on Matrigel-coated polycarbonate filters in modified transwell chambers of 8 μm pore size (Corning, USA), as described previously [29]. Both cells exposed to static and oscillatory flow for 24 h were used for the study. Invaded cells were counted using ImageJ software. The number of invaded cells was calculated as the average of seven random fields in each experiment.

### Statistical analysis

All data obtained in this study were analyzed using GraphPad Prism version 8 (San Diego, CA, USA). qRT-PCR data from tissue specimens were represented as scatter plots. The difference between patients and controls was calculated using one-way analysis of variance (ANOVA). Cell-based analysis was repeated at least thrice. Data obtained are expressed as the mean ± standard deviation (SD) of three repeats. Histoscore from immunohistochemistry in all tissues, mRNA folds in cell studies, and mean fluorescence intensity of confocal images are plotted as bar graphs. Error bars in graphs represent standard deviation. A *p*-value < 0.05 was regarded as statistically significant.

## Results

### Dysregulation of EndMT and cell adhesion markers in cAVM nidus

**Fig. 1:**
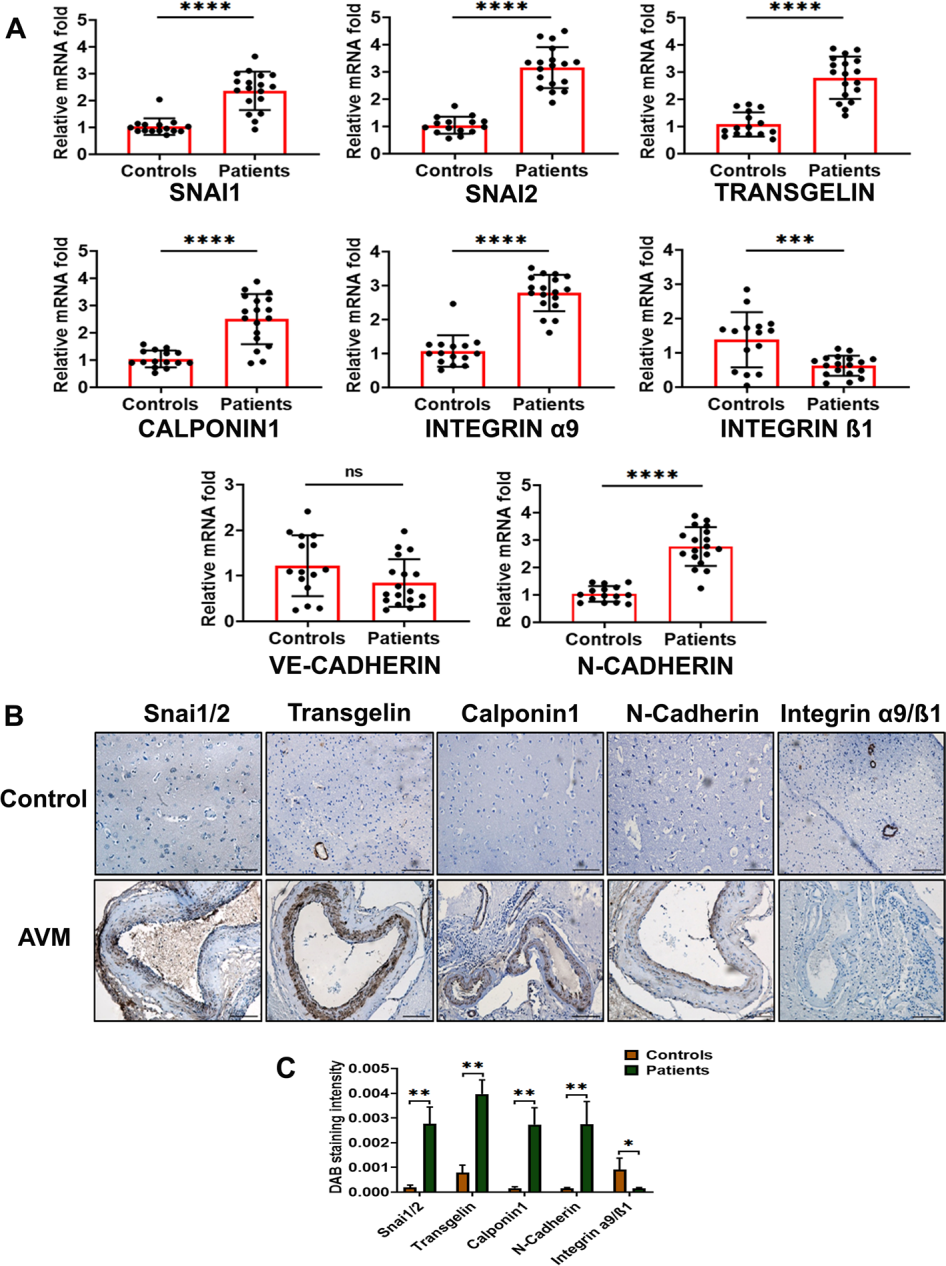
Dysregulation of EndMT and major cell adhesion markers in cerebral AVM nidus. **A** Scatter plots with bars represent the mRNA fold changes of *SNAI1*, *SNAI2*, Calponin 1 (*CNN1*), transgelin (*TAGLN*) and cell adhesion-associated integrin α9 (*ITGA9*), integrin β1 subunits (*ITGB1*), VE-cadherin (*CDH5*), and N-cadherin (*CDH2*) in 18 human cAVM nidi and 15 control brain tissues. *GAPDH* was used as the endogenous control for mRNA fold analysis. Scatter dots represent expression in individual specimens and bars represent mean value. **B** Cerebral AVM nidus consisted of enlarged vessels with medial thickening and intimal hyperplasia. Immunohistochemical staining shows the reduced expression of activated integrin α9/β1 and overexpression of SNAI1/2, Transgelin, Calponin1, and N-cadherin in cAVM vessels compared with control brain vessels. All these EndMT-associated markers were expressed in the intima or neointima of huge AVM nidal vessels (scale bar 100 µm, magnification ×20). **C** Bar graph showing histoscore analysis of SNAI1/2, Transgelin, Calponin1, N-cadherin, and integrin α9/β1 in three random microscopic fields of immunostained sections. Values are mean ± SD. *p < 0.05 versus control tissue, **p < 0.01, ***p < 0.001, ****p < 0.0001. *ns* nonsignificant difference

Our earlier histopathological and immunostaining studies indicated that major vessels in cAVM nidus possess arterial and venous, as well as capillary, endothelial markers [30]. Endothelial cells undergoing mesenchymal transition demonstrate altered morphological features, with subsequent increases in mesenchymal proteins such as transgelin (SM22-α), calponin, etc. We observed that at the mRNA transcript level, *SNAI1*, Slug (*SNAI2*), transgelin (*TAGLN*), and Calponin 1 (*CNN1*) were highly expressed in cAVM samples. *SNAI2* (Slug) had a more prominent expression (3.16 fold) in cAVM than SNAI1 (2.36 fold) when compared with control specimens (Fig. 1A).

During the EndMT process, cell–cell and cell–ECM adhesions of endothelial cells and basement membrane will be disrupted. These molecular changes facilitate the invasiveness of endothelial cells. Hence, we studied the expression of adhesion factors such as cadherins and integrins in cAVM nidus. VE-cadherin (*CDH5*) was downregulated by 0.84 fold in patient samples, while N-cadherin (*CDH2*) mRNA was upregulated (2.76 fold) in cAVMs. Integrin α9 subunit (*ITGA9*) mRNA was overexpressed by 2.78 fold in cAVMs, but integrin β1 (*ITGB1*) mRNA was reduced by 0.63 fold.

Furthermore, we conducted an immunohistochemical staining-based EndMT protein expression and localization analysis in AVM and control tissues. We observed that there was an overexpression of SNAI1/2 in the intimal regions of large vessels in AVMs (Fig. 1B). SNAI1/2 was not expressed in control brain vasculature. There was an approximate 14-fold increase of SNAI1/2 in nidal samples compared with controls, based on histoscore analysis (Fig. 1C). Calponin 1 and transgelin expressed very intensely in both intima and media of cAVM nidal vessels. Transgelin expression was present in small blood vessels of control brains, but Calponin 1 was not expressed in control vasculature.

N-cadherin was localized to the neointimal regions of the nidus tissues and was not observed in control specimens. We also did not observe N-cadherin, a mesenchymal marker, in the medial layer of any of our patient samples. The activated form of integrin, α9/β1, was found to be downregulated in cAVM nidus in contrast to control vasculature.

### Cerebral AVMs express higher levels of NICD3 and NICD4

**Fig. 2:**
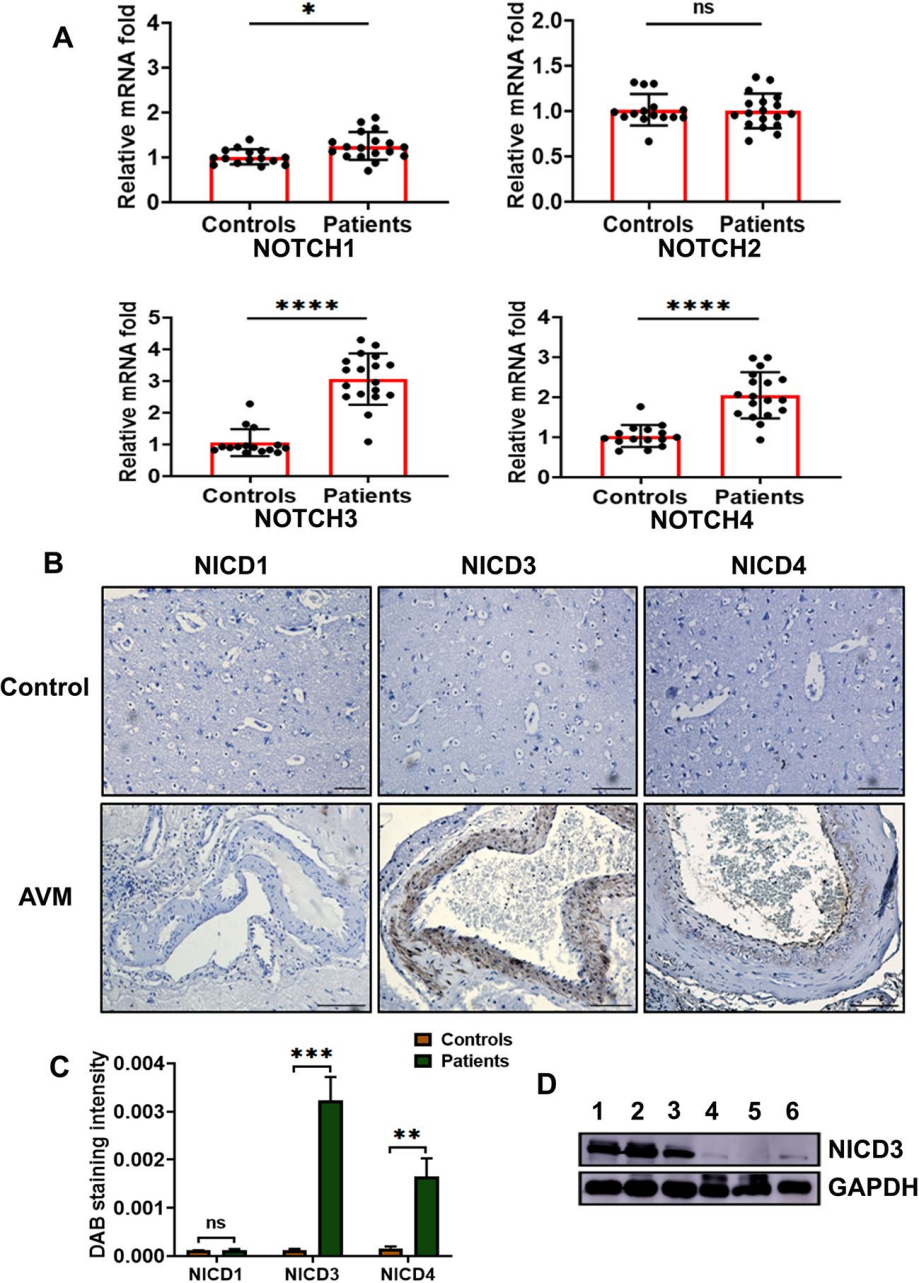
Expression profile of Notch receptors in cerebral AVM nidal vessels. **A** Scatter plots with bar diagrams of mRNA fold changes of *Notch* 1–4 receptors in 18 human cAVM nidi and 15 control brain tissues. *GAPDH* was taken as the endogenous control for quantification. **B** Representative photomicrographs of immunostaining illustrate that Notch intracellular domain (NICD) proteins NICD3 and NICD4 are overexpressed in cAVM compared with control brain vasculature (scale bar 100 µM, magnification ×20). **C** Semiquantitative histoscore analysis shows significant overexpression of NICD3 and NICD4 proteins in cAVMs. **D** Representative western blot of NICD3 protein expressed in three cerebral AVM nidi and three control tissues. GAPDH was considered as the loading control. Lanes 1–3: cAVM nidus, and lanes 4–6: control tissues. Values are mean ± SD. *p < 0.05 versus control tissue, **p < 0.01, ***p < 0.001, ****p < 0.0001. *ns* not significant

We initially studied the expression of all four Notch receptor genes, *Notch* 1–4, in the cAVM nidus. *Notch3* and *Notch4* mRNAs were significantly overexpressed in nidus specimens, compared with control specimens, by approximately three and two fold, respectively (Fig. 2A). *Notch1* was slightly overexpressed in cAVMs (1.26 fold) in comparison with control mRNA. *Notch2* expression levels were statistically insignificant in cAVM nidus.

We specifically investigated the expression of activated Notch receptors i.e., Notch intracellular domains (NICD) in cAVM tissue sections and control brain samples. NICD1 was also included in the immunohistochemical studies considering its reported role in endothelial cells, but none of the control or AVM nidal vessels expressed it [31]. NICD3, the Notch mural variant, was found to be highly expressed and localized to the intima and media of nidal vessels, indicating EndMT (Fig. 2B, C). NICD4 receptor levels in cAVM were lower compared with NICD3 and were seen in a diffused pattern, more toward intima and less in the tunica media of tortuous vessels. Elevated expression of NICD3 in AVM nidus was further corroborated by western blot analysis in three of the control tissues and nidi (Fig. 2D, Additional file 2).

### Oscillatory flow promotes gamma-secretase-dependent Notch receptor activation

**Fig. 3:**
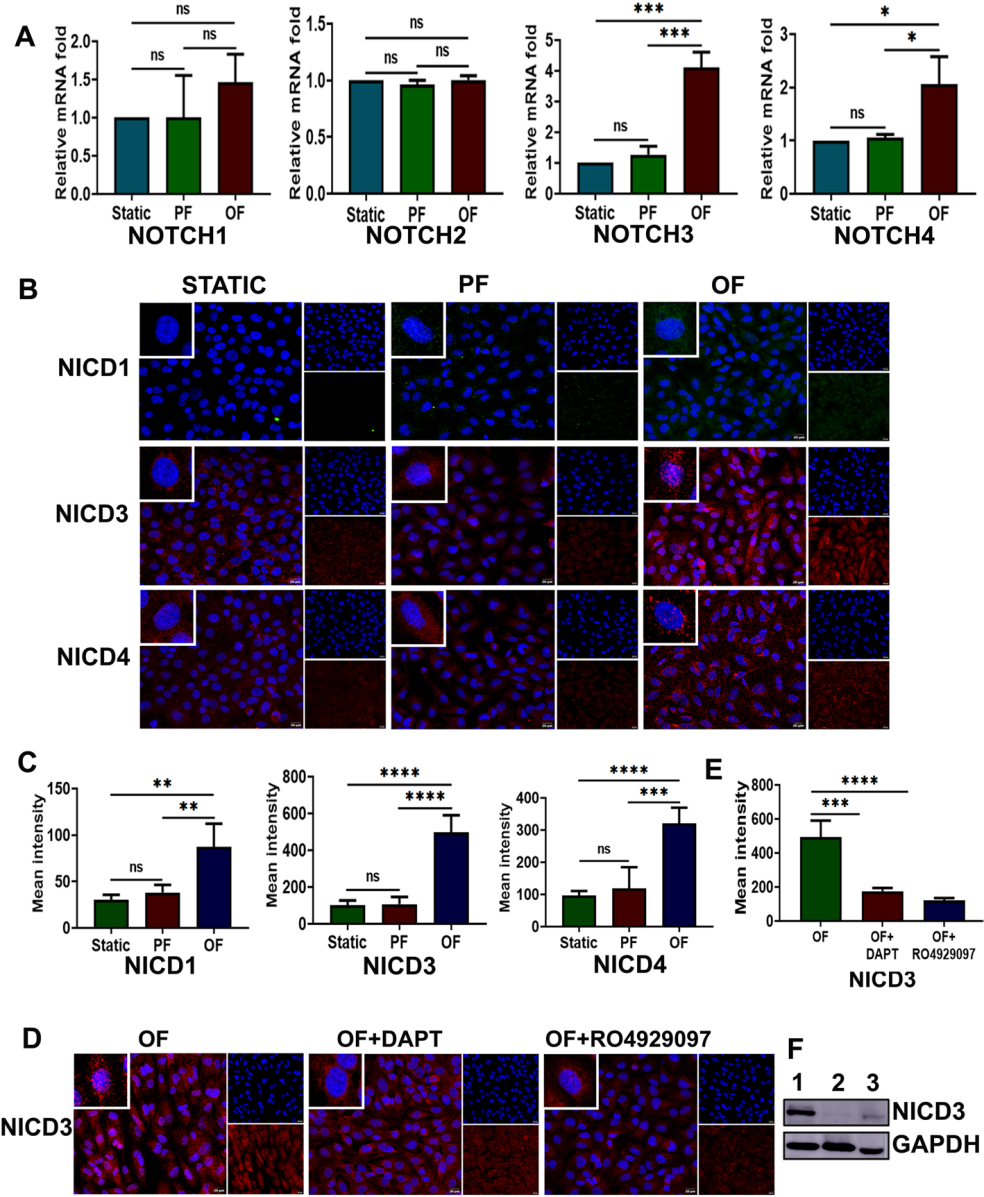
Notch receptor activation by oscillatory fluid flow in human cerebral microvascular endothelial cells (hCMEC/d3). **A** mRNA level expression of Notch receptors upon exposure of hCMEC/d3 to disturbed flow (*n* = 3). *Notch3* and *Notch4* become prominent as endothelial cells are exposed to oscillatory flow for 24 h, while *Notch1* and *Notch2* expressions were not significantly regulated by altered fluid flow. mRNA fold values in parallel and oscillatory flow were calculated relative to the static control. All data were normalized with *GAPDH* expression and are given as relative to static control. **B** hCMEC/d3 exposed to oscillatory flow at 15 dyn/cm^2^ for 24 h resulted in the overexpression of NICD3, which was localized to the nucleus. NICD4 was also overexpressed in cells exposed to oscillatory flow, but cytoplasmic localization was more prominent. NICD1 was very faintly expressed in cells exposed to oscillatory flow, but was detected in mean fluorescence intensity (MFI) analysis. DAPI (blue) was used to counterstain nuclei (scale bar 20 µM, magnification ×40). **C** MFI was plotted as the average fluorescence intensity ± SD of five microscopic fields per flow condition and from three biological replicates. **D**, **E** DAPT and RO4929097 efficiently prevented NICD3 expression in endothelial cells in the presence of continuing altered flow. **F** Representative western blot of NICD3 protein present in proteins isolated from cells exposed to static (lane 3), parallel (lane 2), and oscillatory (lane 1) shear stress conditions. GAPDH was considered as the loading control. PF indicates parallel uniform shear stress and OF represents oscillatory shear stress. *p < 0.05, **p < 0.01, ***p < 0.001, ****p < 0.0001 versus respective static or parallel uniform shear-treated groups. *ns* not significant

To identify whether oscillatory shear stress-dependent Notch receptor activation occurs in endothelial cells, we conducted qRT-PCR-based mRNA analysis and protein immunofluorescence assay in hCMECs exposed to defined flow conditions using a microfluidic flow chamber. Initially, hCMECs were characterized for von Willebrand factor expression (Additional file 1: Fig. S1). We then analyzed the gene expression profile in hCMECs exposed to uniform parallel shear stress (15 dyn/cm^2^) for 24 h. We found that the mRNA expression of *Notch1*–*4* under parallel unidirectional flow conditions did not vary significantly from the expression under static flow conditions (Fig. 3A). Oscillatory flow induced overexpression of *Notch3* (4.11 fold) and *Notch4* (2.06 fold) mRNAs in endothelial cells, compared with static flow. Compared with cells exposed to parallel flow, oscillatory flow resulted in a 3.2 and 1.96 fold increase of *Notch3* and *Notch4*, respectively. Both parallel and oscillatory flow did not induce significant *Notch1* and *Notch2* expression in hCMECs, compared with static flow conditions (Fig. 3A).

Next, we investigated the NICD 1, 3, and 4 protein-level expression and their cellular localization in hCMECs exposed to various flow conditions. There was an overexpression of NICD3 in endothelial cells exposed to oscillatory shear stress compared with parallel flow and static conditions. Oscillatory shear stress altered the subcellular localization of the Notch3 receptor by promoting the nuclear localization of NICD3 (Additional file 1: Fig. S2A). Cells were also found to be elongated with a spindle-like morphology after 24 h of flow. NICD4 expression was marginally elevated, but the localization was predominantly cytoplasmic. NICD1 expression was not significantly observed in cells exposed to any of the three flow conditions, but MFI analysis indicated a slight overexpression in cells exposed to oscillatory flow (Fig. 3B, C). To rule out non-specific staining, we performed negative control assays with secondary antibodies in the absence of primary antibodies (Additional file 1: Fig. S3).

Furthermore, we studied whether oscillatory shear stress directly induces Notch receptor activation by inducing gamma-secretase activity. MTT assay was conducted to decide the optimum DAPT and RO4929097 concentrations (Additional file. 1. Fig. S4). On the basis of the viability offered, hCMECs were treated with 0.5 µM DAPT and 250 nM RO4929097 during the 24 h oscillatory flow exposure. Both inhibitors negatively affected the shear stress response of the gamma-secretase-induced cleavage of the Notch3 receptor in hCMECs (Fig. 3D, Additional file 1: Fig. S2A). However, the efficacy of RO4929097 was superior to DAPT and statistically significant (*p* = 0.0007) (Fig. 3E). Elevated NICD3 protein-level expression in cells exposed to oscillatory flow was further substantiated by western blot analysis (Fig. 3F, Additional file 2).

### Oscillatory flow-induced EndMT requires Notch receptor activation

**Fig. 4:**
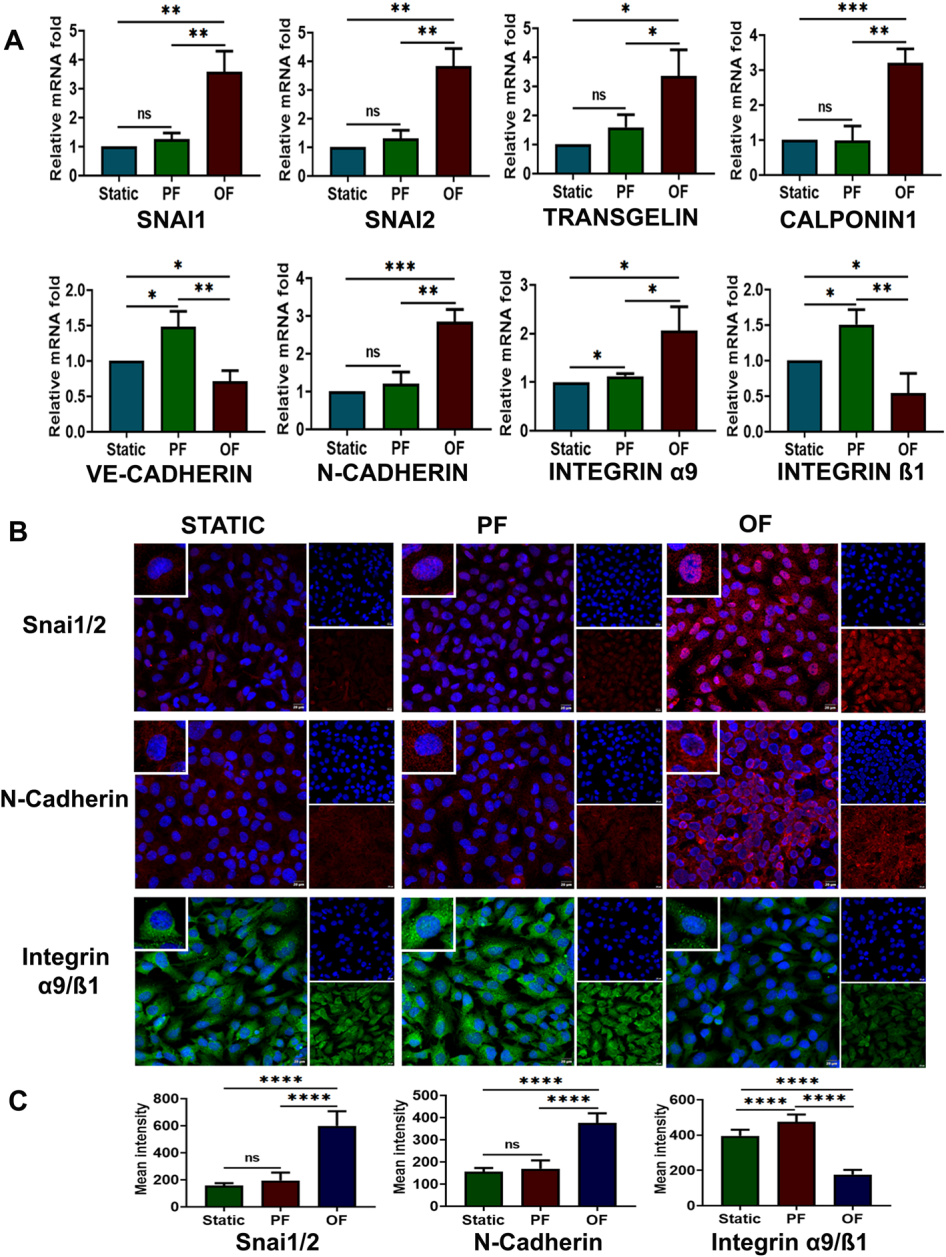
Differential expression of EndMT and cell adhesion markers by oscillatory flow in microvascular endothelial cells. **A** mRNA expression of genes coding for SNAI1, SNAI2, Calponin1, Transgelin, integrin α9 subunit, integrin β1 subunit, VE-cadherin, and N-cadherin upon exposure of hCMEC/d3 to oscillatory flow (*n* = 3). The mRNA expression folds of SNAI1, SNAI2 (Slug), Calponin1, transgelin, integrin α9 subunit, and N-cadherin were significantly higher after exposure to oscillatory flow for 24 h. Integrin β1 subunit and VE-cadherin were found to be downregulated in cells exposed to oscillatory flow. mRNA fold values were calculated relative to static control. All data were normalized with *GAPDH* expression and are given as relative to static control. **B** hCMEC/d3 exposed to oscillatory flow for 24 h increased nuclear SNAI1/2 and N-cadherin expression, while integrin α9/β1 was highly downregulated, indicating active EndMT and reduced cell adhesion among cells exposed to oscillatory flow (scale bar 20 µM, magnification ×40). **C** Mean fluorescence intensity was plotted as the average fluorescence intensity ± SD of five fields per flow condition and from three biological replicates. PF indicates parallel uniform shear stress and OF represents oscillatory shear stress. *p < 0.05, **p < 0.01, ***p < 0.001, ****p < 0.0001 versus respective static or parallel uniform shear-treated groups. *ns* not significant

hCMECs were used to study the loss of endothelial markers and the gain of mesenchymal characteristics in response to oscillatory shear stress. mRNA expression analysis revealed the upregulation of mesenchymal *CNN1* (3.21 fold) and *TAGLN* (3.37 fold) in cells exposed to oscillatory shear stress when compared with static shear stress. The mRNAs of key EndMT markers *SNAI1* and *SNAI2* were also overexpressed by 3.59 and 3.83 fold, respectively, in oscillatory flow-exposed cells (Fig. 4A). As expected, *Slug* expression was marginally higher compared with *SNAI1* in cells exposed to oscillatory flow, corroborating the findings in cAVM.

Immunofluorescence analysis was performed for SNAI1/2 in hCMECs exposed to static, parallel, and oscillatory shear stress. SNAI1/2 was overexpressed and localized to the nucleus in cells exposed to oscillatory flow compared with other flow regimes (Fig. 4B, (Additional file 1. Fig. S2B). MFI analysis indicated a 3.8 and 3.1 fold increase in SNAI1/2 expression, when compared with static and parallel flow, respectively (Fig. 4C).

**Fig. 5:**
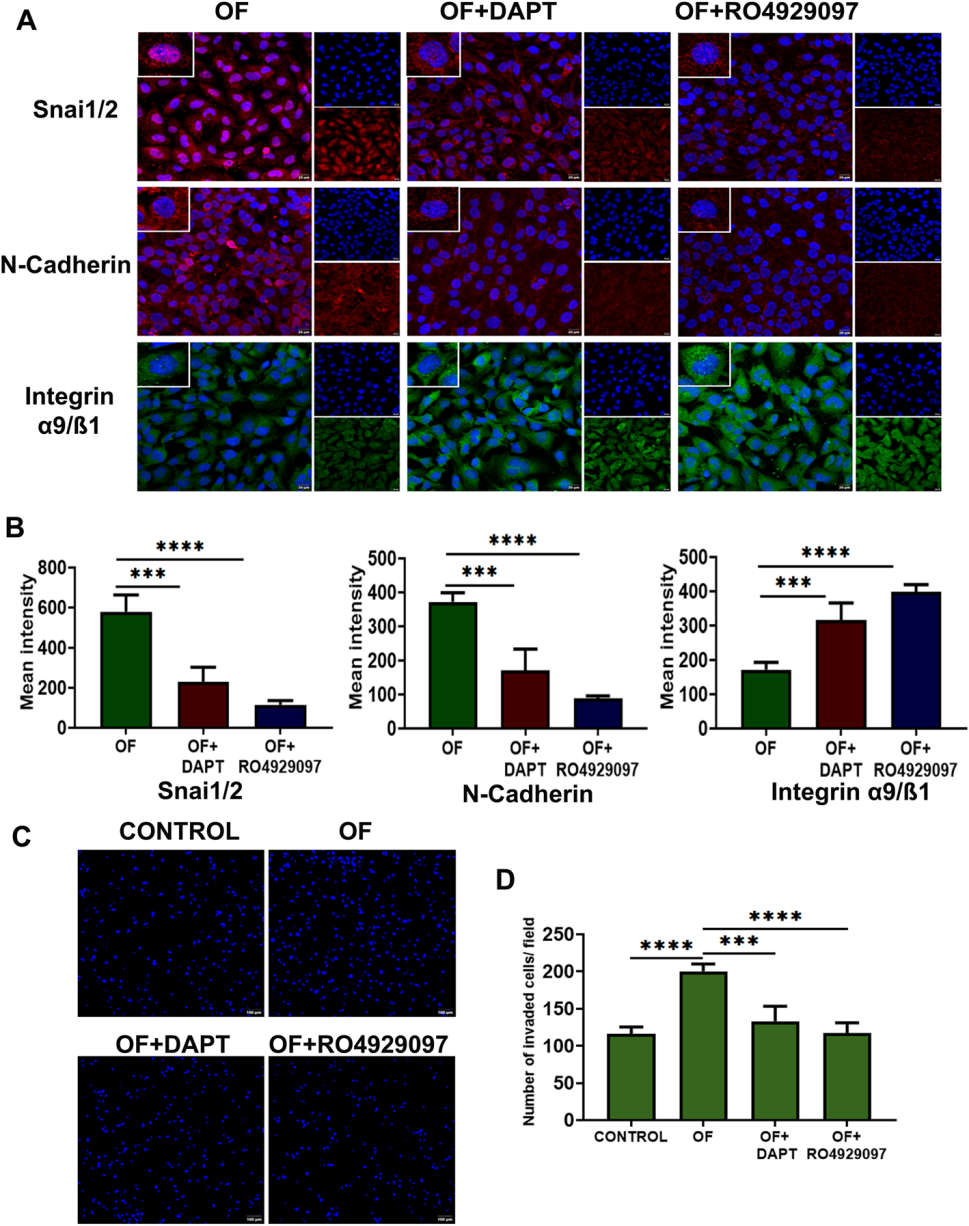
Gamma-secretase inhibitors (GSI) modulate oscillatory shear-induced EndMT and cell invasiveness. **A**, **B** Inhibition of Notch receptor cleavage by 500 nM DAPT and 250 nM RO4929097 prevented the overexpression of SNAI1/2 and N-cadherin in hCMEC/d3 exposed to oscillatory flow for 24 h. Integrin α9/β1 expression was augmented by GSI even in the continuing presence of oscillatory shear stress. EndMT-associated molecular changes were significantly reduced in the presence of RO4929097 (scale bar 20 µM, magnification ×40). **C** Fluorescent microscopic images of invaded cerebral microvascular endothelial cells, with prior exposure to control and oscillatory flows, at the lower surface of the transwell Matrigel-coated filter membrane stained with 5 μg/ml of nuclear stain Hoechst 33342 in the presence and absence of each inhibitor (250 nM RO4929097 and 500 nM DAPT) (scale bar 100 µM, magnification ×10). **D** Invasion assay was done in triplicate, and invaded cells were counted by ImageJ and plotted graphically. As noted in the graph, cells exposed to 24 h oscillatory flow invade faster than normal endothelial cells. The presence of DAPT (*p* < 0.001) and RO4929097 (*p* < 0.0001) effectively reduced the percentage of invaded oscillatory shear-exposed endothelial cells when compared with cells exposed to oscillatory flow alone. OF represents oscillatory shear stress. *p < 0.05, **p < 0.01, ***p < 0.001, ****p < 0.0001 versus respective static or parallel uniform shear-treated groups

To assess the involvement of the Notch pathway in shear stress-induced EndMT in hCMECs, we used GSI DAPT (0.5 µM) and RO4929097 (250 nM) during oscillatory flow and monitored the expression of EndMT factors. Inhibition of Notch receptor cleavage abrogated the downregulation of SNAI1/2 observed during oscillatory stress by approximately 60% with DAPT and 80% with RO4929097 (Fig. 5A, B, Additional file 1: Fig. S2B). Taken together, these data indicate that oscillatory shear stress-induced EndMT requires Notch signaling. Gamma-secretase inhibitor RO4929097 is very efficient in reducing mesenchymal phenotype in endothelial cells during altered flow conditions.

### RO4929097 promotes cell adhesion and reduces invasiveness in cells exposed to oscillatory flow

To corroborate the findings on dysregulation of cell adhesion factors in cAVM tissues, we studied, by qRT-PCR, the mRNA expression of genes coding for endothelial VE-cadherin, mesenchymal N-cadherin, and integrin subunits α9 and β1 in endothelial cells exposed to various flow regimes. N-cadherin (2.85 fold) and integrin α9 (2.06 fold) were upregulated, while VE-cadherin (0.71 fold) and integrin β1 (0.54 fold) were downregulated at mRNA level in cells exposed to oscillatory shear stress (Fig. 4A).

Immunofluorescence assay of these adhesion factors in cells exposed to shear stress conditions indicated that there is an upregulation of N-cadherin in cells exposed to oscillatory shear stress versus cells exposed to static and parallel shear stress. Activated integrin α9/β1 was found significantly reduced in cells exposed to oscillatory flow for 24 h (Fig. 4B, C).

DAPT and RO4929097 significantly reduced the N-cadherin in cells exposed to continuing oscillatory flow. Interestingly, integrin α9/β1 expression was augmented in the presence of DAPT and RO4929097 by 45.5% and 56.7%, respectively, when the MFI of confocal images were analyzed (Fig. 5A, B).

Increased cell invasiveness is an important consequence of EndMT. To study the invasive properties of the hCMECs in response to flow regimes, we used a Matrigel transmembrane invasion assay, where migration of cells toward a source of serum attractant, across a membrane coated with Matrigel, was measured. Prior exposure of cells to oscillatory shear stress for 24 h induced an increase in endothelial invasiveness, which was substantially blocked by both DAPT and RO4929097 (Fig. 5C). RO4929097 effectively prevented cell invasiveness compared with DAPT (Fig. 5D). This finding suggests that pharmacological intervention targeting Notch signaling can effectively reduce blood flow-induced invasion capacity and EndMT of vascular endothelial cells.

## Discussion

Given the recent discovery of EndMT markers in arteriovenous malformations (AVMs) and the recognition of the importance of EndMT in the pathogenesis of brain AVMs, we explored the triggers and mechanisms that activate EndMT in these malformations. We found that EndMT markers SNAI1 and SNAI2, as well as mesenchymal markers transgelin and Calponin1, are strongly expressed in the intima of large vessels of cAVM nidus. We also found evidence for the involvement of Notch signaling in the pathogenesis of cAVMs. Microfluidic-based shear stress studies revealed that oscillatory shear stress induces the expression of Notch3 in cerebral microvascular endothelial cells. Altered shear stress-induced Notch signaling induces N-cadherin that confers higher invasiveness to endothelial cells. There was also a loss of expression of transmembrane adhesion factors, such as integrin α9/β1 and VE-cadherin, in human cAVMs. Earlier studies by others and our group have demonstrated the presence of active angiogenesis and postnatal vascular remodeling in AVMs [1, 30]. We had reported earlier the overexpression of α-SMA in endothelial and subendothelial layers of AVMs [30], thus indicating the activation of EndMT. EndMT in cAVMs was first reported by Yao et al. in 2019 [32]. They demonstrated mesenchymal stem cell markers Sox2 and N-cadherin in the endothelium of large nidal vessels. We report the presence of key EndMT transcriptional factors and mesenchymal factors in the luminal side of large vessels in human cAVM nidus, providing direct evidence for EndMT.

Factors that induce EndMT and contribute to the pathophysiological process of cAVM are currently not known. Studies indicate that SMAD-dependent TGF-β signaling is not activated in brain AVMs [3]. However, Xu et al. reported that KRAS mutants develop EndMT via the classic SMAD pathway, independent of the Notch signaling. Interestingly, in our studies, we have not found any tissue-level expression of pSMAD2 and pSMAD1/5/9 in our patient samples (Additional file 1: Fig. S5). Hence, we assume that EndMT in these patients occurs independent of KRAS signaling [33]. Among several factors that could contribute to the EndMT process, altered hemodynamics due to tangled arteriovenous shunt is plausibly an important aspect. The interplay between EndMT and biomechanical force in regulating endothelial dysfunction and vascular remodeling in AVMs is hypothesized [34], but the underlying mechanisms are unclear.

Our fluid shear stress-based experiments suggest that flow disturbances can induce higher SNAI1/2 expression and mesenchymal protein levels in human cerebral microvascular endothelial cells. How is altered shear stress coupled with the EndMT process? In our earlier studies, we observed high expression of Notch ligands, such as Dll4, and Notch target genes, such as Hey2 and Ephrin B2, in AVM nidus [30]. Notch receptors, once activated by respective ligands, are cleaved by membrane protease gamma-secretase to generate an intracellular domain of the Notch receptor (NICD). NICD then translocates into the nucleus and activates downstream signaling inducing transcriptional inducer Hey and repressor Hes proteins. Murphy, et al. have reported that canonical Notch signaling exemplified augmented Hes1 expression in brain AVMs [14]. Notably, Notch signaling is a major mechanotransductive pathway in endothelial cells [11]. It has been observed that increased vascular wall shear stress activates Notch1 and 4 signaling in rat AVM models [35]. Notch1 and Notch4 receptors are mainly detected in endothelial cells [36]. However, NICD1 expression in endothelial cells exposed to oscillatory flow conditions, and even laminar flow conditions, was minimal, which was surprising. Mack, et al. observed around a two-fold increase of Notch1 in human aortic endothelial cells when exposed to 12 h of laminar flow and was reduced after 24 h of exposure [37]. Our studies were single timepoint assays, and at 24 h of laminar parallel flow exposure, hCMECs expressed 1.25 fold Notch1 protein, on the basis of fluorescence intensity analysis.

NICD4 was expressed in the cytoplasm of cells exposed to oscillatory conditions. Interestingly, NICD3, a mural cell marker [38], was significantly overexpressed in endothelial cells in response to oscillatory shear stress, indicating extensive EndMT. We adopted a comparatively longer flow exposure period of 24 h to understand the chronic exposure effects, rather than Notch expression on short-term shear stress exposures. We also exposed hCMECs to high shear stress (23 dyn/cm^2^) (Additional file 1: Fig. S6), but the nuclear localization of NICD3 was observed to be minimal.

Slug, rather than SNAI1, was found to be more expressed in cAVM samples and cells exposed to oscillatory flow in our studies. It can be explained by the fact that Slug is a direct target of the Notch pathway in endothelial cells [39]. Furthermore, we used two GSI, i.e., DAPT and RO4929097, to confirm the association between Notch receptor activation and elevated SNAI1 and Slug expression under oscillatory flow conditions. The expression of EndMT markers was abrogated in the presence of these Notch inhibitors, even under continued altered fluid flow conditions.

Corroborating our cell-based observations, there was a very low expression of activated NICD1 and 2 in cAVM tissues. Notch1 and Notch4, but not Notch2, were previously reported in brain AVMs [40]. ZheGhe et al. noted that NICD1 action is prominent in inducing a proangiogenic state in postnatal disease progression in brain AVMs [31]. A significant NICD1 expression was not found in cAVM nidus at protein levels in our study, even though upregulated Notch1 receptor mRNA was noted in cAVM nidus. Whether the reduced NICD1 protein expression in cAVMs is due to the lack of ligand binding, or post-transcriptional modification of Notch mRNA, is yet to be studied. Unlike a similar study on tissue-level Notch receptors by Sandra Hill-Felberg, et al. [40], we did not observe NICD1 expression in control vessels. Our study demonstrates that NICD3 and NICD4 are the two most highly expressed Notch receptors in cAVM nidus and suggests their significance in disease pathogenesis.

AVM lesions grow and undergo remodeling postnatally [41]. These cellular processes are linked to proliferation, migration, and invasion. EndMT is associated with a higher invasive cellular phenotype [42]. We observed downregulation of the active form of cell–ECM adhesion factor and integrin α9/β1, and upregulation of N-cadherin in cAVM nidus, as well as in cells exposed to oscillatory flow. Cells exposed to altered flow, as seen in cAVM nidus, predominantly have N-cadherins, which are comparatively less stable and dynamic adhesion factors compared with VE-cadherins, which act as a repressor for invasiveness. Masia et al. demonstrated a direct interaction of NICD and its target gene Hes1 with the regulatory regions of N-cadherin and α9-integrin subunit genes [43]. Their coordinating regulation may influence the VE-cadherin/N-cadherin balance in cells undergoing EndMT. DAPT and RO4929097-mediated Notch inhibition normalized the expression of cell adhesion factors in the continuing presence of oscillatory flow; they also reduced cell invasiveness. RO4929097 counteracted the effect of disturbed shear stress more efficiently than DAPT. RO4929097 was highly effective in reverting Notch-mediated endothelial plasticity, as opposed to DAPT. Moreover, RO4929097 was more potent on a dose–response basis than DAPT.

NICD expression analysis in surgical samples cannot confirm its role in the causation of cAVMs. Moreover, Notch-based AVM pathogenesis is further complicated by lncRNAs and miRNAs, which have functional interplay with this signaling cascade [44]. Yet, the elevated NICDs undoubtedly indicate disease aggressiveness in cAVMs. Notch signaling is thus a plausible target for a noninvasive management strategy in patients with cAVMs. Gamma-secretase inhibitors of the Notch pathway are currently under clinical trial for patients with various cancers [45]. Notch has significant physiological roles in normal cells, such as arteriovenous specification, mechanotransduction, etc. [46]. However, to achieve long-term therapeutic effects in AVMs, prolonged Notch inhibition may not be necessary. We have demonstrated the efficacy of RO4929097 in reversing EndMT using in vitro experiments, and thus provided evidence for future animal model-based studies and clinical trials. Further studies are warranted to evaluate the effects of pan-Notch inhibition in preclinical models, as well as to develop specific inhibitors for Notch3 that may help in reducing pan-Notch inhibition-based side effects.

## Conclusions

Our data demonstrate the presence of Notch receptors and EndMT markers in human cAVM nidus. To our knowledge, this is the first report indicating that altered flow-induced Notch signaling causes EndMT in cAVMs. We provide evidence for the direct role of Notch signaling in augmenting cell invasiveness in endothelial cells exposed to disturbed blood flow.

## Supplementary Information

**Additional file 1: Fig. S1.** Characterization of hCMEC/d3 with von Willebrand factor. **Fig. S2.** Quantification of nuclear versus cytosolic localization of **A** NICD3 and **B** SNAI1/2 in hCMECs exposed to various flow conditions and treatments. **Fig. S3.** Immunofluorescence with secondary antibodies alone. **Fig. S4.** Cell viability studies of DAPT and RO4929097 by MTT reduction assay. **Fig. S5.** Immunohistochemical localization of pSMAD2 and pSMAD1/5/9 in control and cAVM tissues. **Fig. S6**. Immunofluorescence assay of NICD3 in hCMECs exposed to higher shear stress. **Table S1.** Primers used for quantitative real-time PCR. **Table S2.** Summary of source and dilutions of antibodies used for immunohistochemistry (IHC) and immunofluorescence (IF) assays.

**Additional file 2. **Uncut gel images of western blots included in Figs. 2D and 3F.

## Data Availability

All data sets generated and analyzed to support the conclusion of this study are included in this published article and its additional files.

## Abbreviations

cAVM: Cerebral arteriovenous malformations
EndMT: Endothelial mesenchymal transition
hCMEC: Human cerebral microvascular endothelial cells
IBIDI: Integrated Bio Diagnostics
vWF: Von Willebrand factor
qRT-PCR: Quantitative real-time PCR
