# Supplementary material for: Oscillatory shear stress modulates Notch-mediated endothelial mesenchymal plasticity in cerebral arteriovenous malformations
Source: Cell Mol Biol Lett. 2023 Mar 18;28:22. doi: 10.1186/s11658-023-00436-x (PMC10024393; doi:10.1186/s11658-023-00436-x)
Supplement: Supplementary file 1 — Additional file 1: Fig. S1. Characterization of hCMEC/d3 with von Willebrand factor. Fig. S2. Quantification of nuclear versus cytosolic localization of A NICD3 and B SNAI1/2 in hCMECs exposed to various flow conditions and treatments. Fig. S3. Immunofluorescence with secondary antibodies alone. Fig. S4. Cell viability studies of DAPT and RO4929097 by MTT reduction assay. Fig. S5. Immunohistochemical localization of pSMAD2 and pSMAD1/5/9 in control and cAVM tissues. Fig. S6. Immunofluorescence assay of NICD3 in hCMECs exposed to higher shear stress. Table S1. Primers used for quantitative real-time PCR. Table S2. Summary of source and dilutions of antibodies used for immunohistochemistry (IHC) and immunofluorescence (IF) assays. [file 11658_2023_436_MOESM1_ESM.doc]

**Oscillatory shear stress modulates Notch-mediated endothelial mesenchymal plasticity in cerebral arteriovenous malformations.**

Karthika CL^1#^, Vani Venugopal^1#^, Sreelakshmi BJ^1^, Krithika S^1^, Jaya Mary thomas^1^, Mathew Abraham^2^, Kartha CC^3^, Arumugam Rajavelu^4^, Sumi S^1^*

**
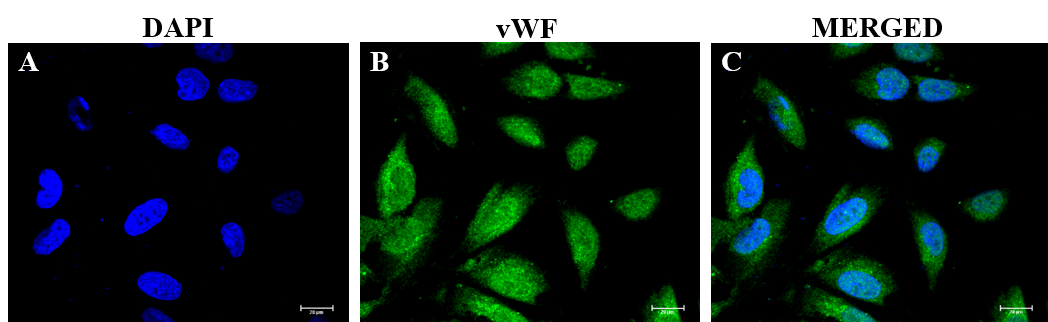
**

**Additional file 1. Fig. S1. Characterization of hCMEC/d3 with von Willebrand factor.** (A) Nuclear staining with DAPI (blue), (B) anti vWF antibody (1:100, DAKO A0082), (C) Merged image. The positive staining obtained with von Willebrand factor (vWF) antibody suggested the presence of Weibel- Palade bodies, secretion granules present in endothelial cells. Magnification- 40X, Scale bar- 20μm

**
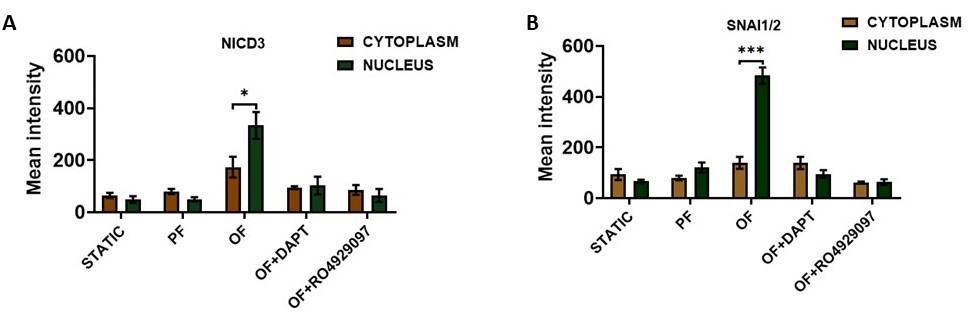
**

**Additional file 1. Fig. S2. Quantification of nuclear *vs* cytosolic localization of (A) NICD3 and (B) SNAI1/2 in hCMECs exposed to various flow conditions and treatments.** Nuclear localization of NICD3 and SNAI1/2 are prominent in cells exposed to oscillatory flow (OF) compared to parallel flow (PF) and static flow. Gamma-secretase inhibitors, DAPT and RO4929097 effectively prevented the expression and nuclear localization of NICD3 and SNAI1/2 even under ensuing oscillatory flow. Values are the mean fluorescent intensity in nuclei and cytoplasm of 3 random fields. * denotes P<0.05, *** P<0.001


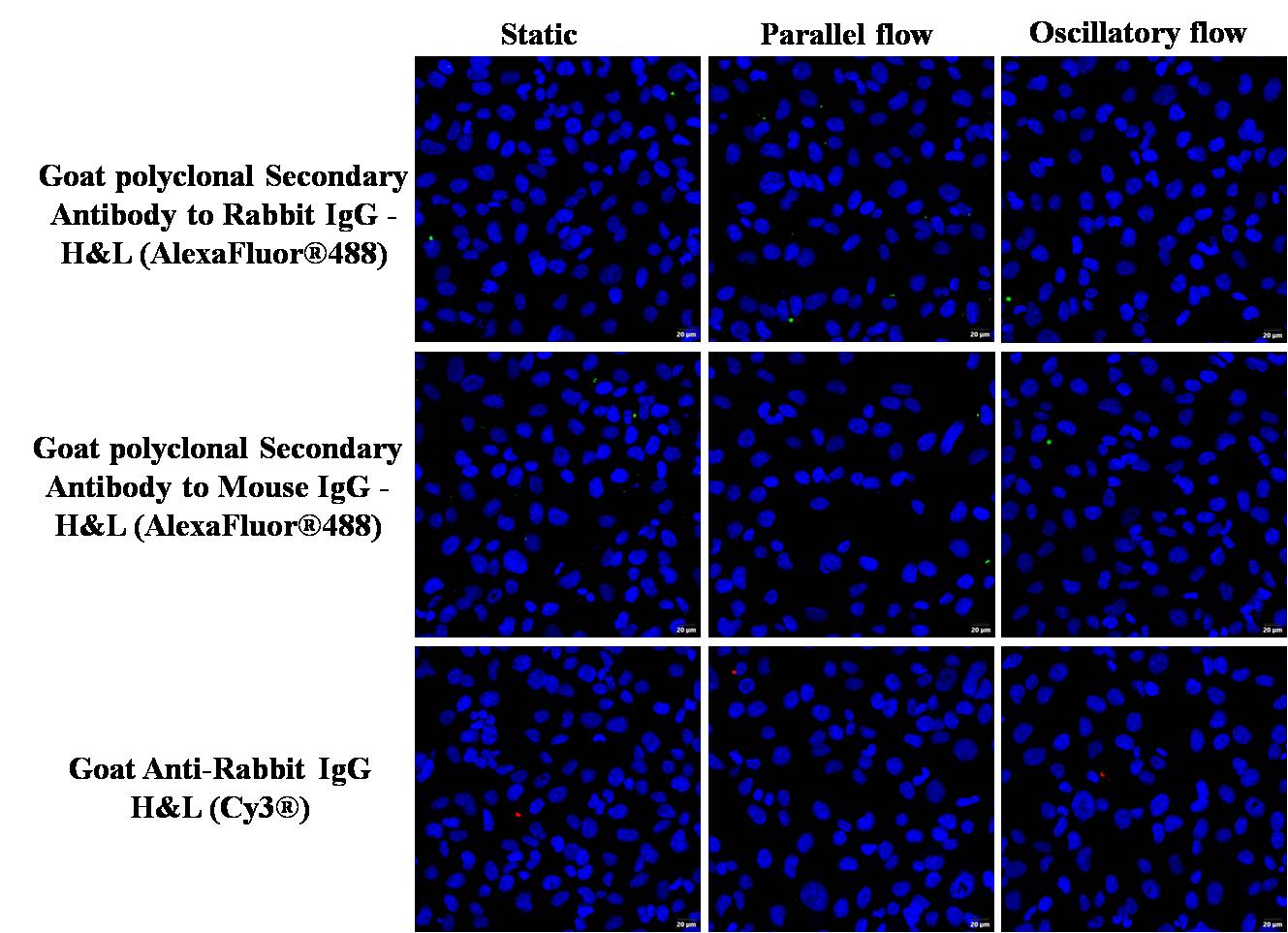


**Additional file 1. Fig. S3. Immunofluorescence with secondary antibodies alone**. hCMEC/d3 exposed to static, parallel flow and oscillatory flow conditions and stained with secondary antibodies alone demonstrates the absence of non-specific staining. Magnification- 40X, Scale bar- 20μm

**
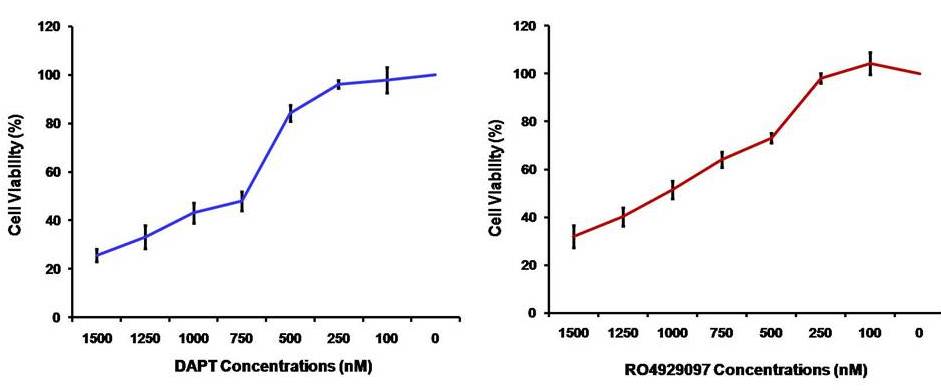
**

**Additional file 1. Fig. S4. Cell viability studies of DAPT and RO4929097 by MTT reduction assay.** hCMEC/D3 treated with gamma-secretase inhibitors DAPT and RO4929097 for 24 h. The lowest concentration with acceptable viability (500nM for DAPT) and (250nM for RO4929097) was selected for further cell-based inhibitor assays

**
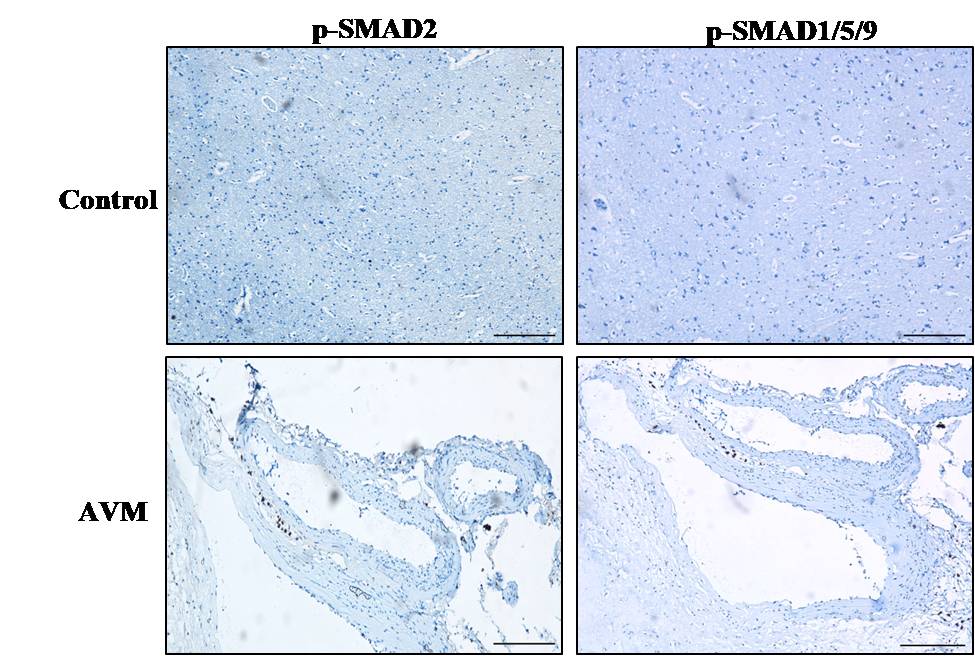
**

**Additional file 1. Fig. S5. Immunohistochemical localization of p-SMAD2 and p-SMAD1/5/9 in control and cAVM tissues.**  Lack of p-SMAD2 (Novus biological, NBP2-66797, Dilution 1:50) and p-SMAD1/5/9 expression (Cell signaling Technology, 13820T, Dilution 1:50) in human cAVM nidus and control brain tissues, as demonstrated by complete absence of DAB staining. Goat anti-rabbit IgG H&L (HRP) at a dilution of 1:100 was used as the secondary antibody (Abcam, ab97051) Magnification-10X, Scale bar-100µm.

**
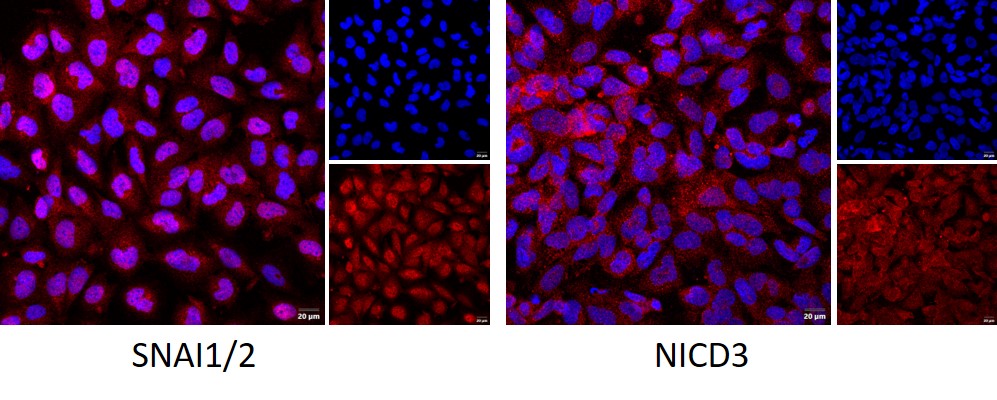
**

**Additional file 1. Fig. S6. Immunofluorescence assay of NICD3 in hCMEC/d3 exposed to higher shear stress**. Exposure of cells to shear stress at 23 dyn/cm^2^ for 24 h resulted in the increased expression of NICD3. Yet the nuclear localization of NICD3 in cells was minimal when compared to oscillatory shear stress (Figure 3B). DAPI (blue) was used to counterstain nuclei. Scale bar 20µM, magnification 40x.

**Additional file 1. Table S1. Primers used for quantitative real-time PCR**

| **Gene** | **Primer sequence 5’ 3’** | **AT (°C)** |
| --- | --- | --- |
| SNAI1 | F- AATCCAGAGTTTACCTTCCAGCAGC  R- AGCCTTTCCCACTGTCCTCATCT | 61 |
| SNAI2 | F- GAACTCACACGGGGGAGAAGCCT  R-GCTACACAGCAGCCAGATTCCTCA | 61 |
| CALPONIN1 | F- ATGGCGAAGACGAAAGGAAACAAG  R-CTGGCTGGCTCCTTTGTTGGTG | 61 |
| TRANSGELIN | F- AATGGCGTGATTCTGAGCAAGCTG  R- ATCTCCACGGTAGTGCCCATCATTC | 61 |
| N - CADHERIN | F- GCAGATAGCCCGGTTTCATTTGAG  R-AGGGCATTGGGATCGTCAGCA | 63 |
| VE - CADHERIN | F- GACCAGGACTTTGACTTGAGCCA  R- CGGGGCTGTGGGGTCAGTATC | 61 |
| INTEGRIN ALPHA9 | F-TGCTGCTCTGGAAGATGGGCT  R-TGGTGTGGCAGCTCACTGGT | 61 |
| INTEGRIN BETA1 | F-AGCTGGTCTCTAAATTGCCGGTGA  R-AGTGTTGTGGGATTTGCACGGG | 61 |
| NOTCH1 | F-GCCGTAGATGACCTGGGCAAGT  R-CGTCCAGCAGCCTCACGATGT | 61 |
| NOTCH2 | F- ATGCGAGTGTGTCCCAGGCTA  R- CTCTTCACAGAGTAGGCCCCGAGT | 57 |
| NOTCH3 | F-CAGGATAGCAAGGAGGAGACCCC  R-CTCCTGCTCTTCTTGGACCCCG | 61 |
| NOTCH4 | F-CCAGGACAACAGGGAGCAGACG  R-TGCGGGGGCACGCTTACT | 61 |
| GAPDH | F-CCAGGCGCCCAATACGACCAA  R-TTCTTTTGCGTCGCCAGCCGA | 60 |

**AT denotes annealing temperature, F forward, R reverse**

**Additional file 1. Table S2. Summary of source, dilutions of antibodies used for immunohistochemistry (IHC) and immunofluorescence (IF) assays**

| **Antibody** | **Source** | **Manufacturer, Cat. No.** | **IF**  **dilution** | **IHC**  **dilution** |
| --- | --- | --- | --- | --- |
| SNAI1/2 | Rabbit | Abcam, ab180714 | 1:100 | 1:50 |
| CALPONIN1 | Rabbit | Abcam, ab46794 | - | 1:100 |
| TRANSGELIN | Mouse | Sigma-Aldrich, MABT167 | - | 1:100 |
| N - CADHERIN | Rabbit | Abcam, ab18203 | 1:100 | 1:100 |
| INTEGRIN ALPHA9 + BETA1 | Mouse | Abcam, ab27947 | 1:100 | 1:50 |
| NOTCH1 | Mouse | Novus Biologicals, NB100-78486SS | 1:100 | 1:100 |
| NOTCH3 | Rabbit | Abcam, ab23426 | 1:100 | 1:100 |
| NOTCH4 | Rabbit | Novus Biologicals, NB100-93551SS | 1:100 | 1:100 |
| vWF | Rabbit | Dako, A0082 | 1:100 | - |
| Goat Anti-Rabbit IgG H&L (HRP) | Goat | Abcam, ab97051 | - | 1:100-1:200 |
| Rabbit anti- mouse IgG H&L (HRP) | Rabbit | Abcam, ab97046 | - | 1:100-1:200 |
| Goat polyclonal Secondary Antibody to Rabbit IgG -H&L (AlexaFluor®488) | Goat | Abcam, ab150077 | 1:200 | - |
| Goat Anti-Rabbit IgG H&L (Cy3®) preadsorbed | Goat | Abcam, ab6939 | 1:200 | - |
| Goat polyclonal Secondary Antibody to Mouse IgG -H&L (AlexaFluor®488) | Goat | Abcam, ab150113 | 1:200 | - |
