# Supplementary figures and images for: Oscillatory shear stress modulates Notch-mediated endothelial mesenchymal plasticity in cerebral arteriovenous malformations
Source: Cell Mol Biol Lett. 2023 Mar 18;28:22. doi: 10.1186/s11658-023-00436-x (PMC10024393; doi:10.1186/s11658-023-00436-x)

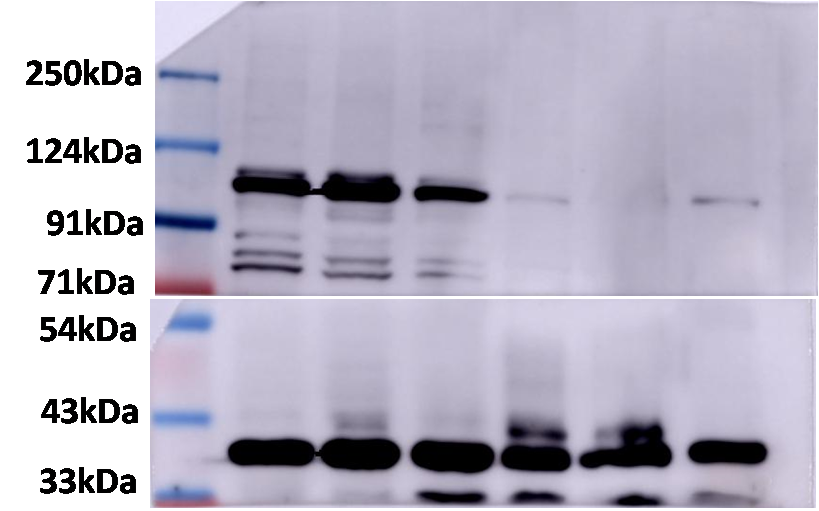


Uncut gel image for Figure 2D


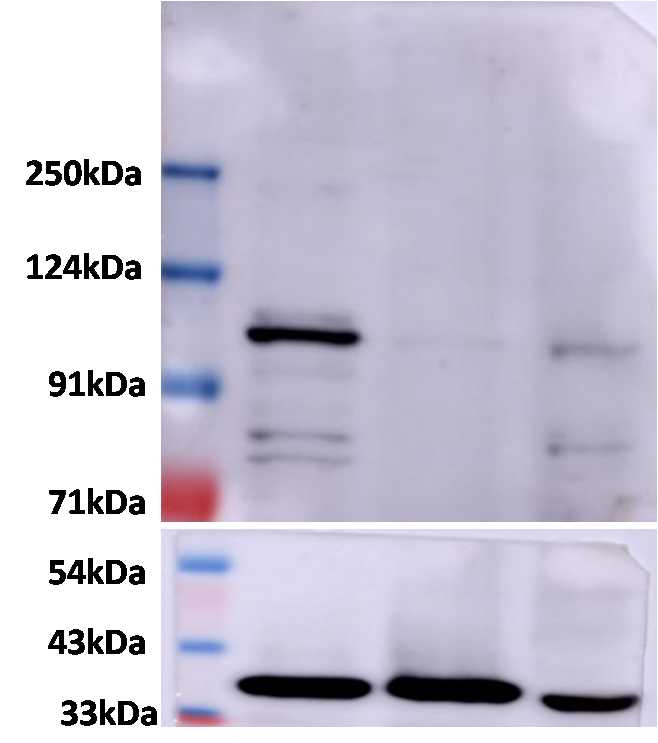


**Uncut gel image for Figure 3F**

Supplement: Supplementary file 2 — Additional file 2. Uncut gel images of western blots included in Figs. 2D and 3F. [file 11658_2023_436_MOESM2_ESM.docx]
